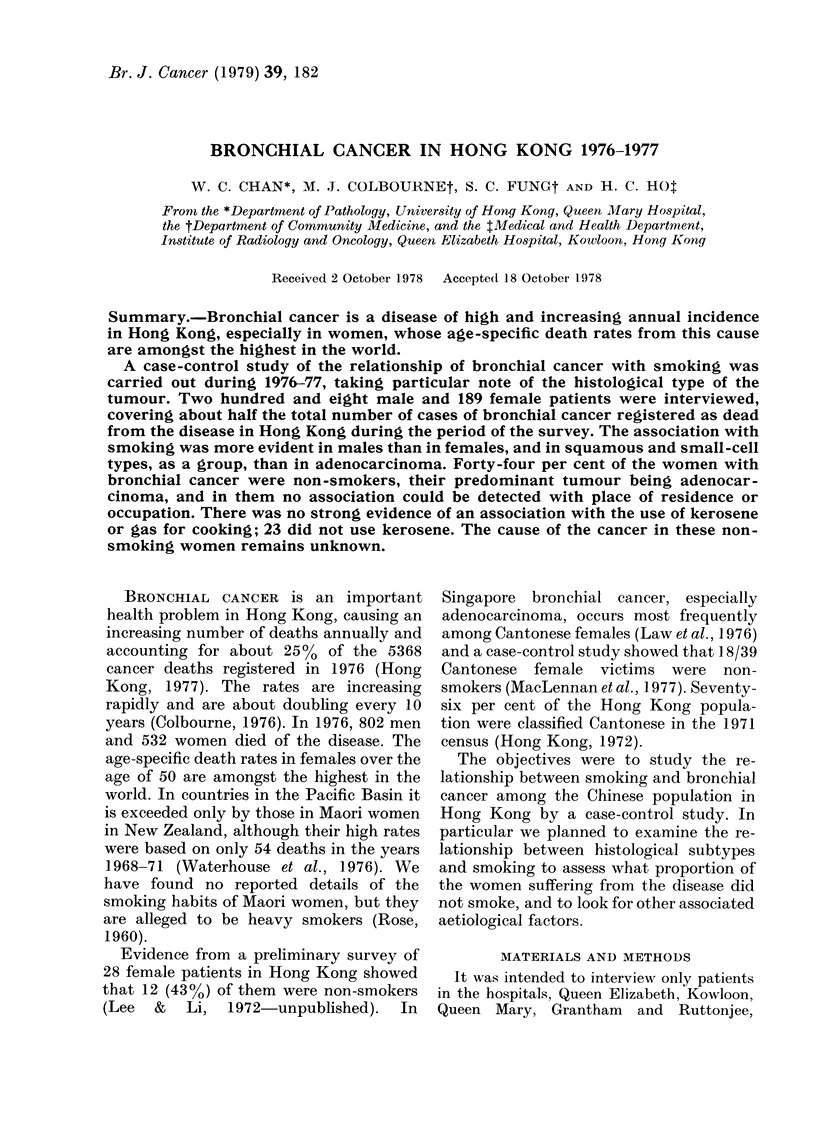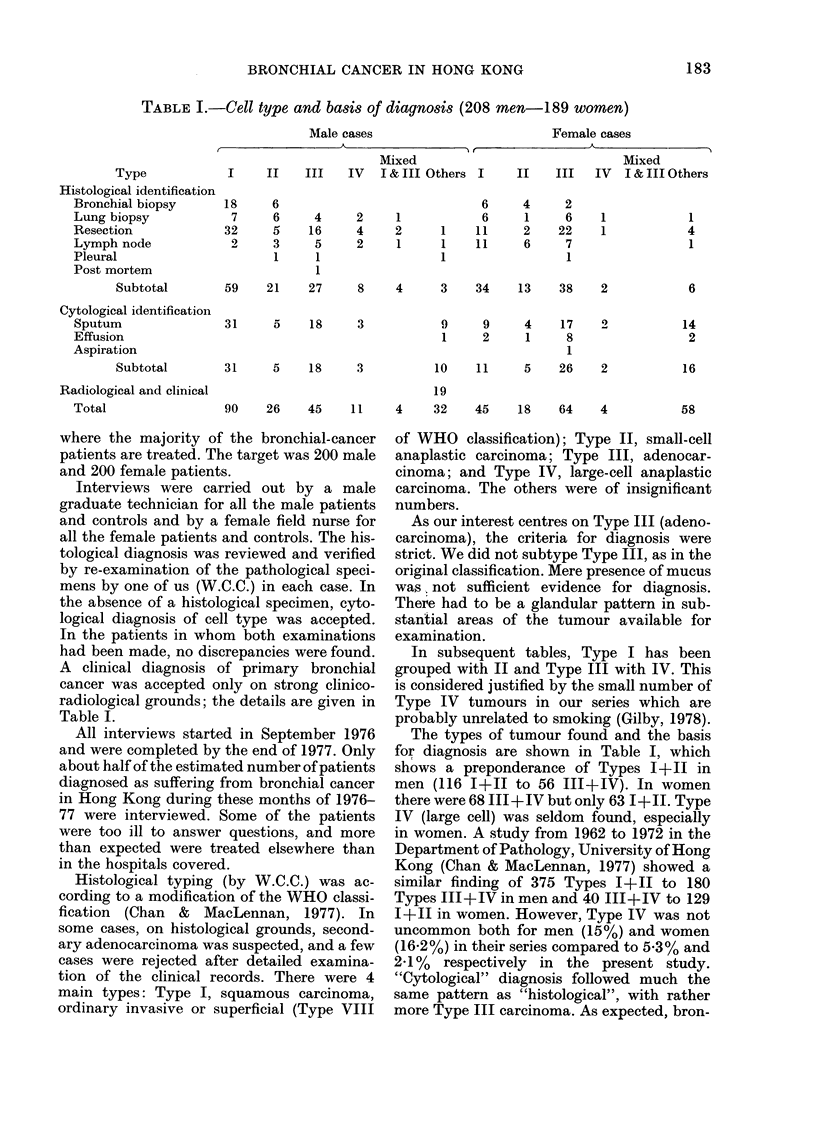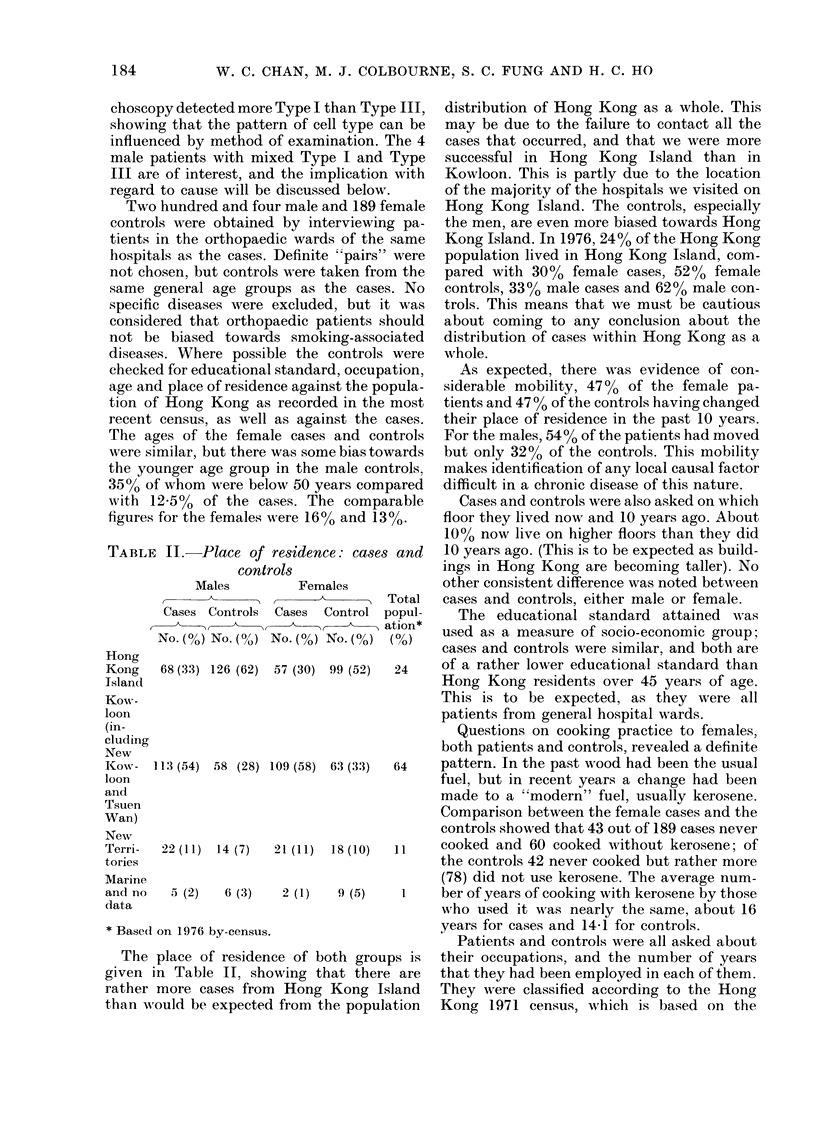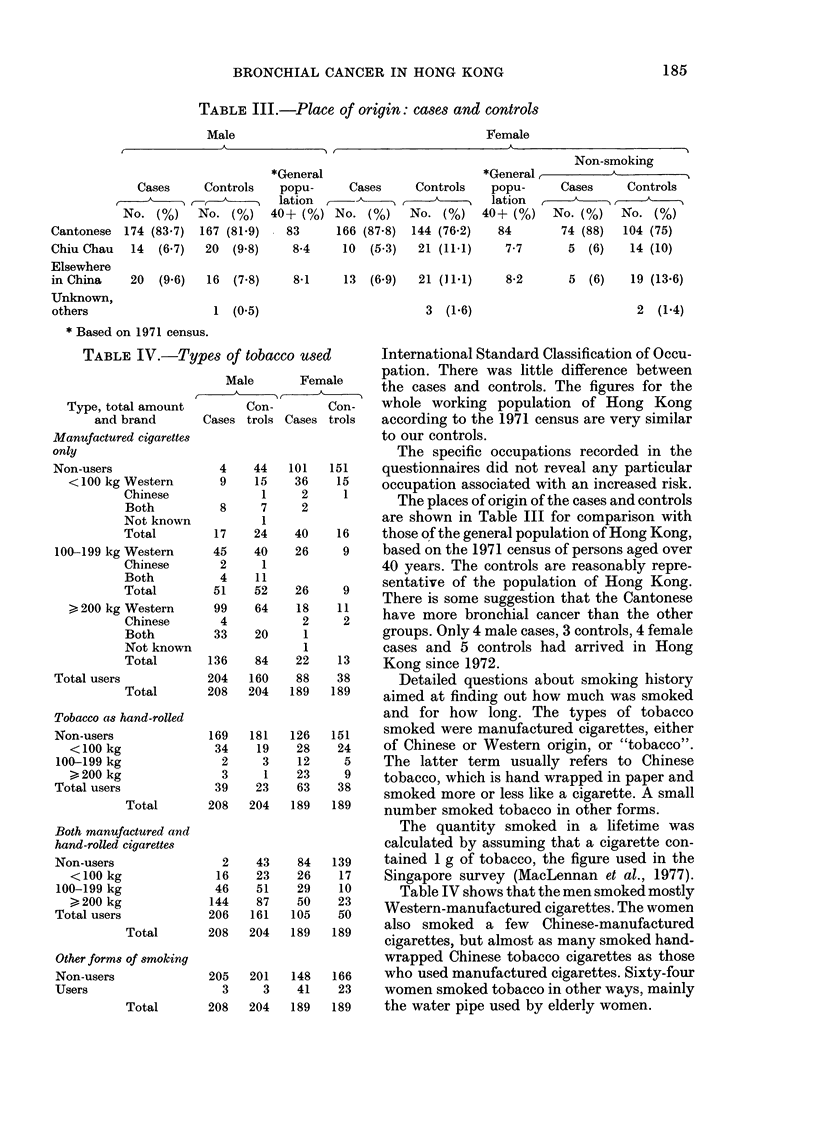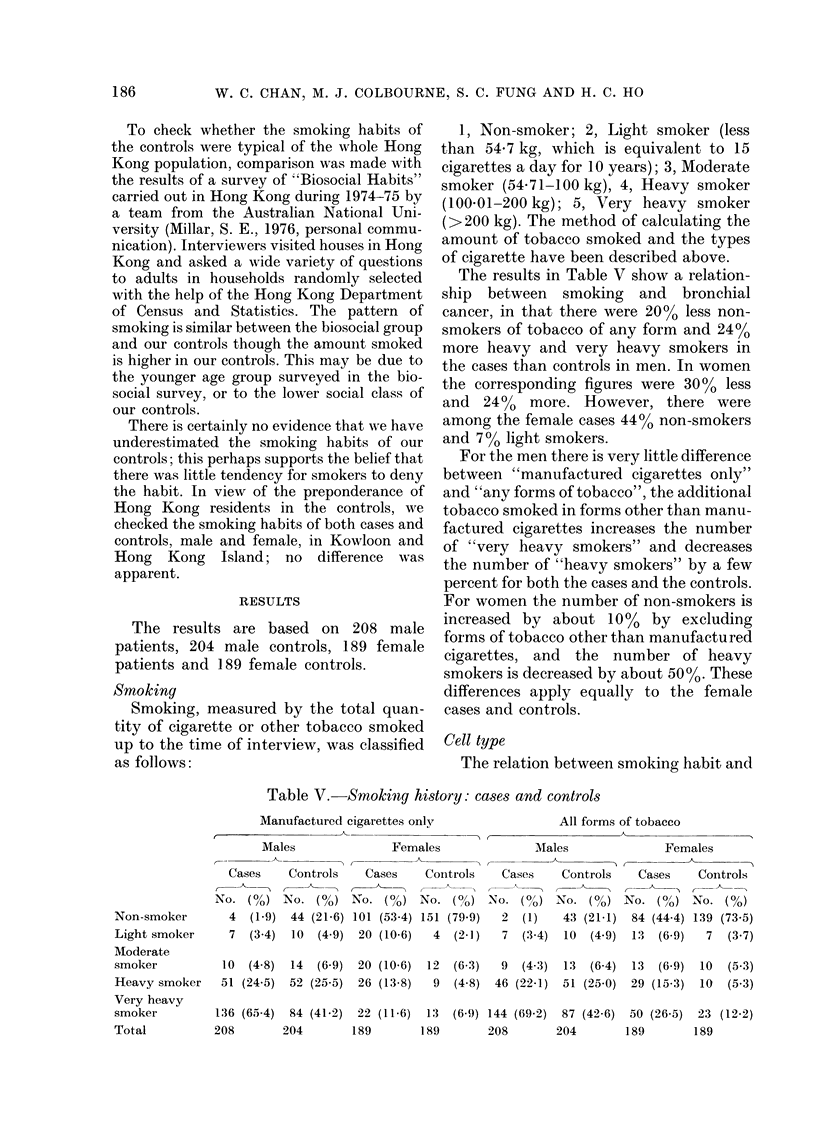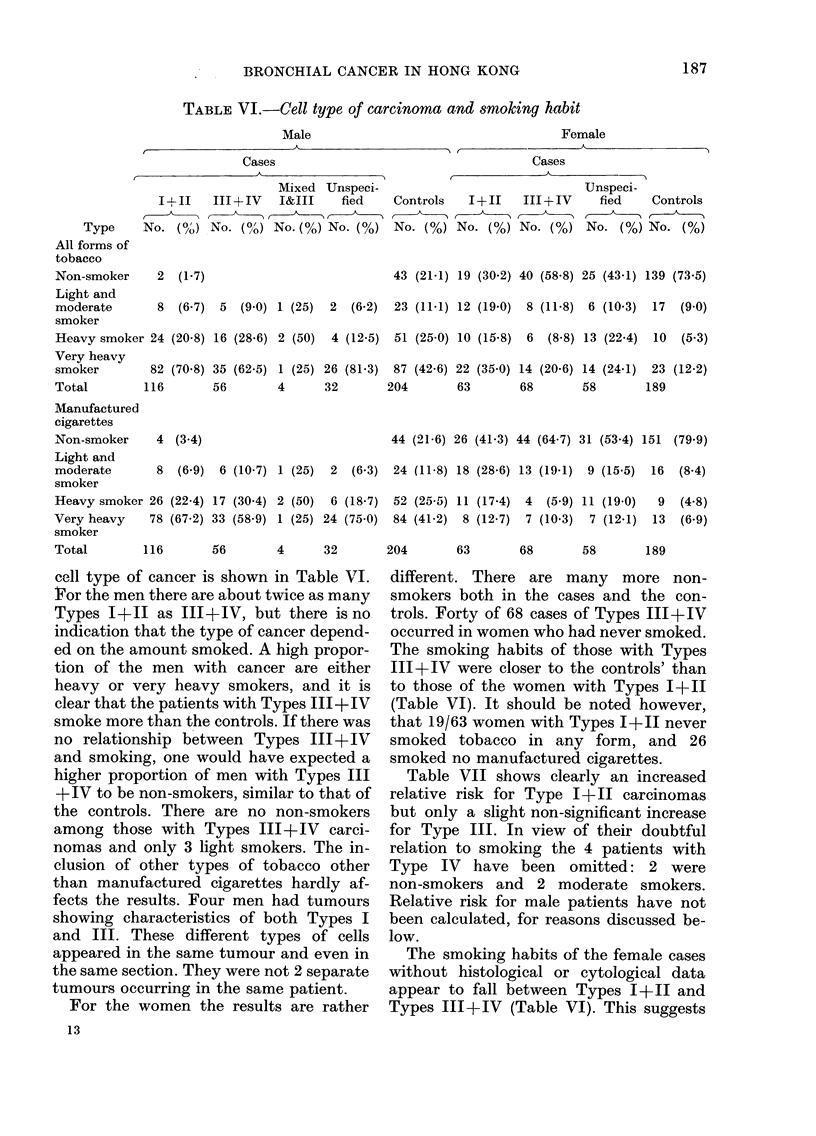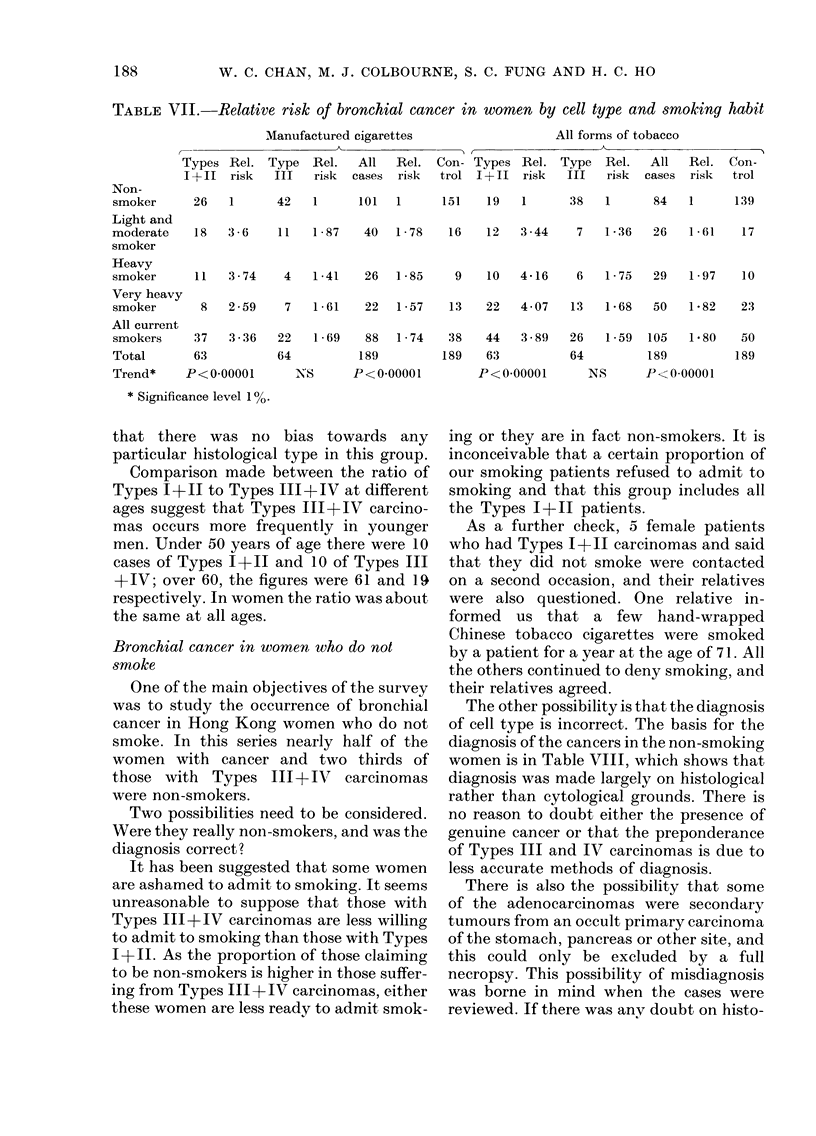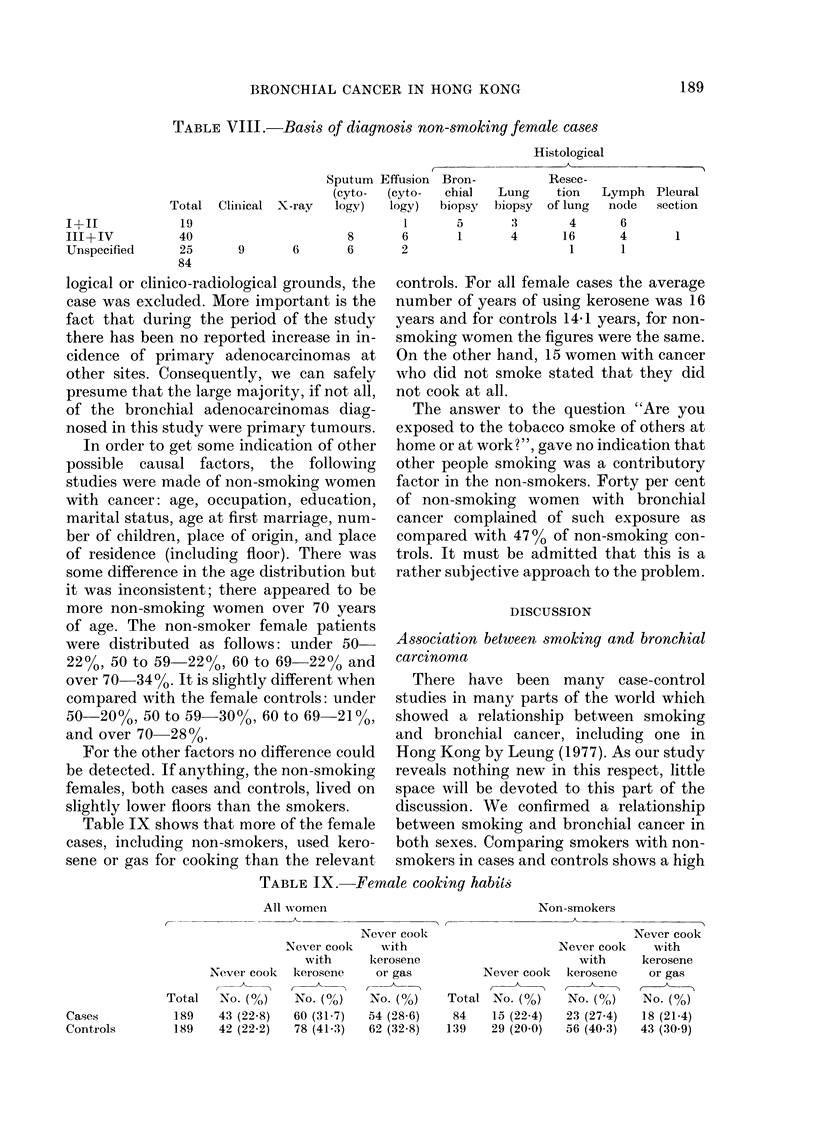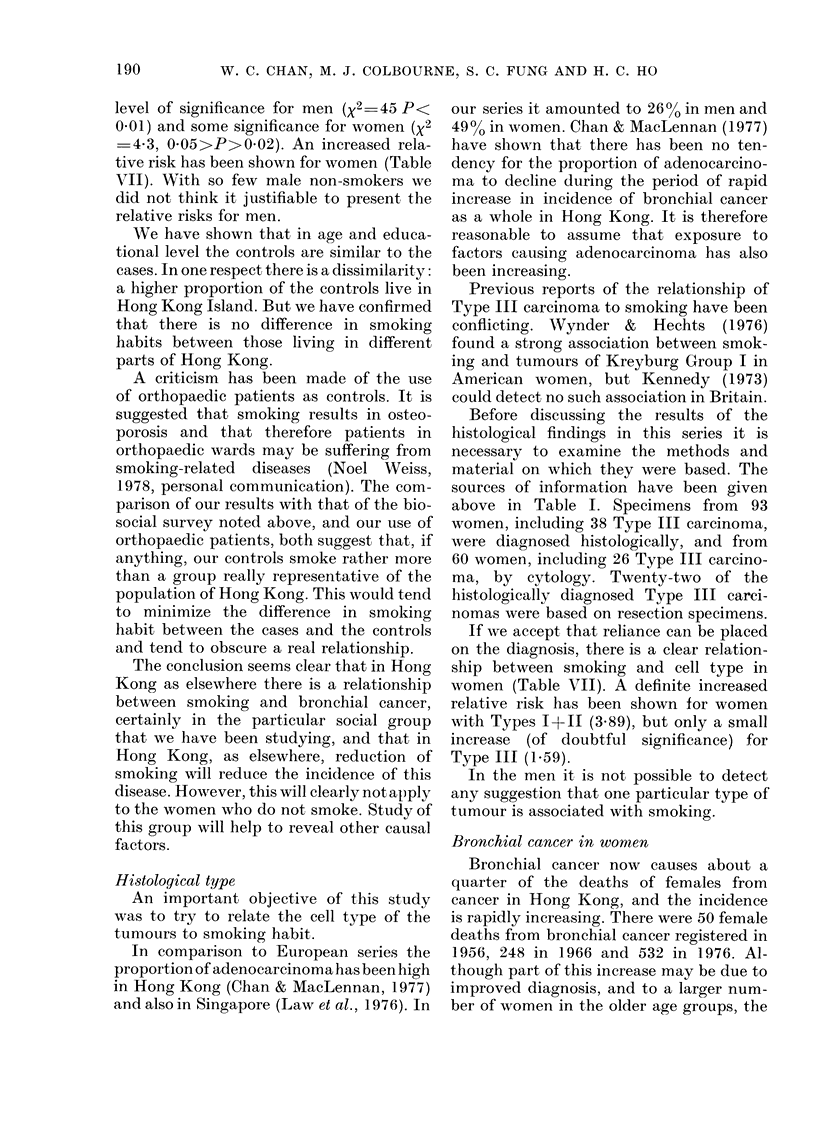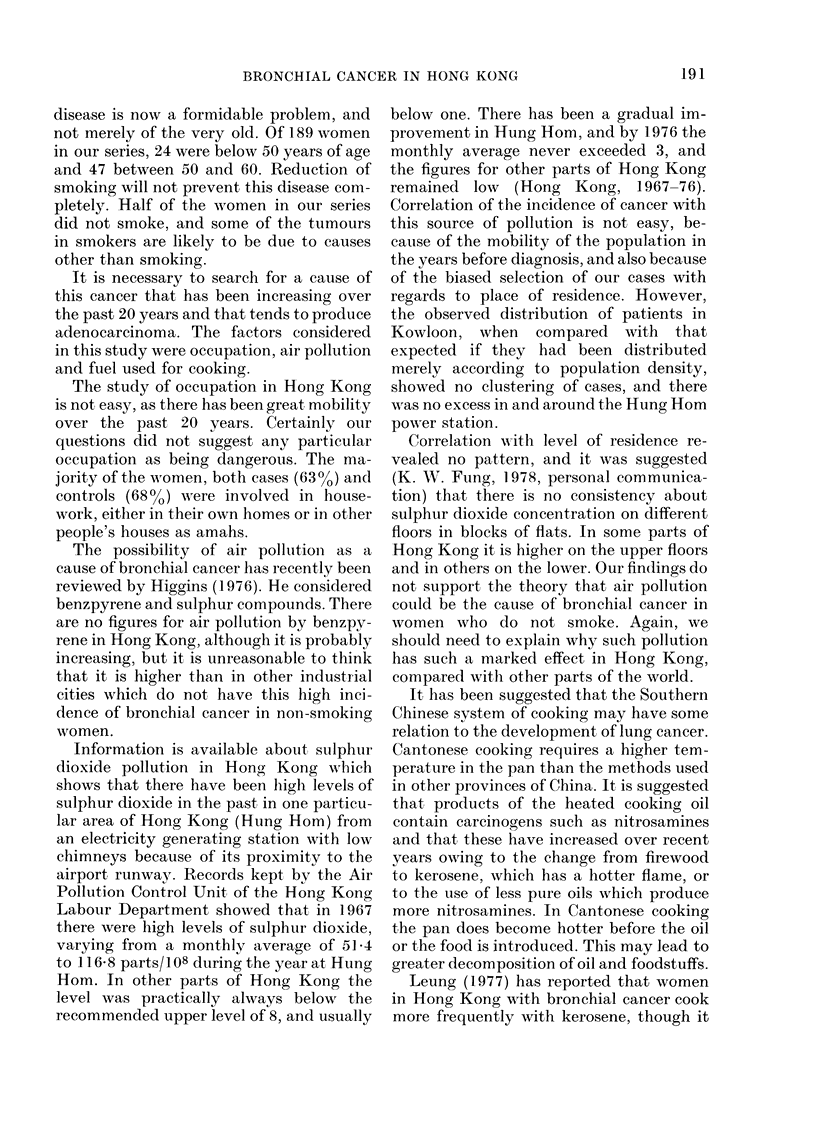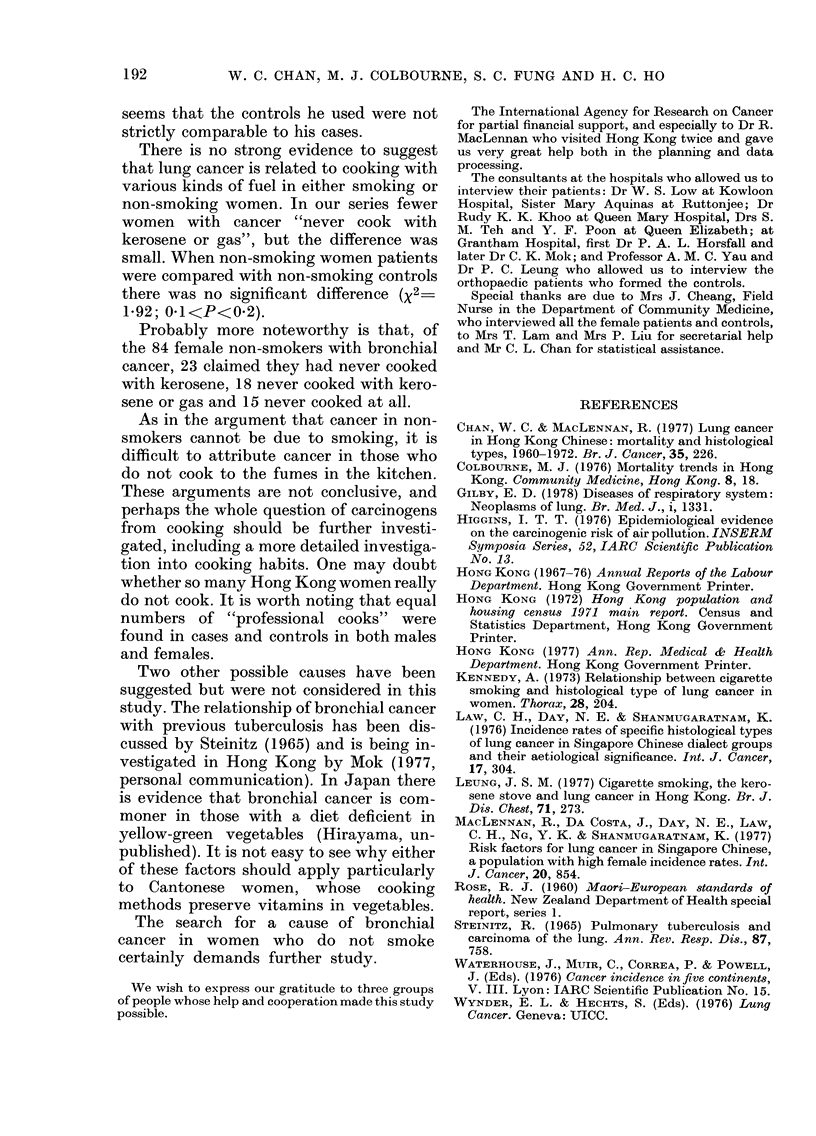# Bronchial cancer in Hong Kong 1976-1977.

**DOI:** 10.1038/bjc.1979.29

**Published:** 1979-02

**Authors:** W. C. Chan, M. J. Colbourne, S. C. Fung, H. C. Ho

## Abstract

Bronchial cancer is a disease of high and increasing annual incidence in Hong Kong, especially in women, whose age-specific death rates from this cause are amongst the highest in the world. A case-control study of the relationship of bronchial cancer with smoking was carried out during 1976--77, taking particular note of the histological type of the tumour. Two hundred and eight male and 189 female patients were interviewed, covering about half the total number of cases of bronchial cancer registered as dead from the disease in Hong Kong during the period of the survey. The association with smoking was more evident in males than in females, and in squamous and small-cell types, as a group, than in adenocarcinoma. Forty-four per cent of the women with bronchial cancer were non-smokers, their predominant tumour being adenocarcinoma, and in them on association could be detected with place of residence or occupation. There was no strong evidence of an association with the use of kerosene or gas for cooking; 23 did not use kerosene. The cause of the cancer in these non-smoking women remains unknown.


*Br. J. Cancer* (1979) **39**, 182

### BRONCHIAL CANCER IN HONG KONG 1976–1977

W. C. CHAN*, M. J. COLBOURNE†, S. C. FUNG† AND H. C. HO‡

*From the *Department of Pathology, University of Hong Kong, Queen Mary Hospital, the †Department of Community Medicine, and the ‡Medical and Health Department, Institute of Radiology and Oncology, Queen Elizabeth Hospital, Kowloon, Hong Kong*

Received 2 October 1978 Accepted 18 October 1978

**Summary.—Bronchial cancer is a disease of high and increasing annual incidence in Hong Kong, especially in women, whose age-specific death rates from this cause are amongst the highest in the world.**

**A case-control study of the relationship of bronchial cancer with smoking was carried out during 1976–77, taking particular note of the histological type of the tumour. Two hundred and eight male and 189 female patients were interviewed, covering about half the total number of cases of bronchial cancer registered as dead from the disease in Hong Kong during the period of the survey. The association with smoking was more evident in males than in females, and in squamous and small-cell types, as a group, than in adenocarcinoma. Forty-four per cent of the women with bronchial cancer were non-smokers, their predominant tumour being adenocarcinoma, and in them no association could be detected with place of residence or occupation. There was no strong evidence of an association with the use of kerosene or gas for cooking; 23 did not use kerosene. The cause of the cancer in these non-smoking women remains unknown.**

BRONCHIAL CANCER is an important health problem in Hong Kong, causing an increasing number of deaths annually and accounting for about 25% of the 5368 cancer deaths registered in 1976 (Hong Kong, 1977). The rates are increasing rapidly and are about doubling every 10 years (Colbourne, 1976). In 1976, 802 men and 532 women died of the disease. The age-specific death rates in females over the age of 50 are amongst the highest in the world. In countries in the Pacific Basin it is exceeded only by those in Maori women in New Zealand, although their high rates were based on only 54 deaths in the years 1968–71 (Waterhouse *et al.*, 1976). We have found no reported details of the smoking habits of Maori women, but they are alleged to be heavy smokers (Rose, 1960).

Evidence from a preliminary survey of 28 female patients in Hong Kong showed that 12 (43%) of them were non-smokers (Lee & Li, 1972—unpublished). In Singapore bronchial cancer, especially adenocarcinoma, occurs most frequently among Cantonese females (Law *et al.*, 1976) and a case-control study showed that 18/39 Cantonese female victims were non-smokers (MacLennan *et al.*, 1977). Seventy-six per cent of the Hong Kong population were classified Cantonese in the 1971 census (Hong Kong, 1972).

The objectives were to study the relationship between smoking and bronchial cancer among the Chinese population in Hong Kong by a case-control study. In particular we planned to examine the relationship between histological subtypes and smoking to assess what proportion of the women suffering from the disease did not smoke, and to look for other associated aetiological factors.

### MATERIALS AND METHODS

It was intended to interview only patients in the hospitals, Queen Elizabeth, Kowloon, Queen Mary, Grantham and Ruttonjee,

TABLE I.—*Cell type and basis of diagnosis* (208 *men*—189 *women*)

| Type | Male cases I | II | III | IV | Mixed I & III | Others | Female cases I | II | III | IV | Mixed I & III | Others |
|---|---|---|---|---|---|---|---|---|---|---|---|---|
| Histological identification | | | | | | | | | | | | |
| Bronchial biopsy | 18 | 6 | | | | | 6 | 4 | 2 | | | |
| Lung biopsy | 7 | 6 | 4 | 2 | 1 | | 6 | 1 | 6 | 1 | | 1 |
| Resection | 32 | 5 | 16 | 4 | 2 | 1 | 11 | 2 | 22 | 1 | | 4 |
| Lymph node | 2 | 3 | 5 | 2 | 1 | 1 | 11 | 6 | 7 | | | 1 |
| Pleural | | 1 | 1 | | | 1 | | | 1 | | | |
| Post mortem | | | 1 | | | | | | | | | |
| Subtotal | 59 | 21 | 27 | 8 | 4 | 3 | 34 | 13 | 38 | 2 | | 6 |
| Cytological identification | | | | | | | | | | | | |
| Sputum | 31 | 5 | 18 | 3 | | 9 | 9 | 4 | 17 | 2 | | 14 |
| Effusion | | | | | | 1 | 2 | 1 | 8 | | | 2 |
| Aspiration | | | | | | | | | 1 | | | |
| Subtotal | 31 | 5 | 18 | 3 | | 10 | 11 | 5 | 26 | 2 | | 16 |
| Radiological and clinical | | | | | | 19 | | | | | | |
| Total | 90 | 26 | 45 | 11 | 4 | 32 | 45 | 18 | 64 | 4 | | 58 |

where the majority of the bronchial-cancer patients are treated. The target was 200 male and 200 female patients.

Interviews were carried out by a male graduate technician for all the male patients and controls and by a female field nurse for all the female patients and controls. The histological diagnosis was reviewed and verified by re-examination of the pathological specimens by one of us (W.C.C.) in each case. In the absence of a histological specimen, cytological diagnosis of cell type was accepted. In the patients in whom both examinations had been made, no discrepancies were found. A clinical diagnosis of primary bronchial cancer was accepted only on strong clinico-radiological grounds; the details are given in Table I.

All interviews started in September 1976 and were completed by the end of 1977. Only about half of the estimated number of patients diagnosed as suffering from bronchial cancer in Hong Kong during these months of 1976–77 were interviewed. Some of the patients were too ill to answer questions, and more than expected were treated elsewhere than in the hospitals covered.

Histological typing (by W.C.C.) was according to a modification of the WHO classification (Chan & MacLennan, 1977). In some cases, on histological grounds, secondary adenocarcinoma was suspected, and a few cases were rejected after detailed examination of the clinical records. There were 4 main types: Type I, squamous carcinoma, ordinary invasive or superficial (Type VIII of WHO classification); Type II, small-cell anaplastic carcinoma; Type III, adenocarcinoma; and Type IV, large-cell anaplastic carcinoma. The others were of insignificant numbers.

As our interest centres on Type III (adenocarcinoma), the criteria for diagnosis were strict. We did not subtype Type III, as in the original classification. Mere presence of mucus was not sufficient evidence for diagnosis. There had to be a glandular pattern in substantial areas of the tumour available for examination.

In subsequent tables, Type I has been grouped with II and Type III with IV. This is considered justified by the small number of Type IV tumours in our series which are probably unrelated to smoking (Gilby, 1978).

The types of tumour found and the basis for diagnosis are shown in Table I, which shows a preponderance of Types I+II in men (116 I+II to 56 III+IV). In women there were 68 III+IV but only 63 I+II. Type IV (large cell) was seldom found, especially in women. A study from 1962 to 1972 in the Department of Pathology, University of Hong Kong (Chan & MacLennan, 1977) showed a similar finding of 375 Types I+II to 180 Types III+IV in men and 40 III+IV to 129 I+II in women. However, Type IV was not uncommon both for men (15%) and women (16·2%) in their series compared to 5·3% and 2·1% respectively in the present study. "Cytological" diagnosis followed much the same pattern as "histological", with rather more Type III carcinoma. As expected, bronchoscopy detected more Type I than Type III, showing that the pattern of cell type can be influenced by method of examination. The 4 male patients with mixed Type I and Type III are of interest, and the implication with regard to cause will be discussed below.

Two hundred and four male and 189 female controls were obtained by interviewing patients in the orthopaedic wards of the same hospitals as the cases. Definite "pairs" were not chosen, but controls were taken from the same general age groups as the cases. No specific diseases were excluded, but it was considered that orthopaedic patients should not be biased towards smoking-associated diseases. Where possible the controls were checked for educational standard, occupation, age and place of residence against the population of Hong Kong as recorded in the most recent census, as well as against the cases. The ages of the female cases and controls were similar, but there was some bias towards the younger age group in the male controls, 35% of whom were below 50 years compared with 12·5% of the cases. The comparable figures for the females were 16% and 13%.

TABLE II.—*Place of residence: cases and controls*

| | Males | | Females | | Total population* |
|---|---|---|---|---|---|
| | Cases | Controls | Cases | Control | |
| | No. (%) | No. (%) | No. (%) | No. (%) | (%) |
| Hong Kong Island | 68 (33) | 126 (62) | 57 (30) | 99 (52) | 24 |
| Kowloon (including New Kowloon and Tsuen Wan) | 113 (54) | 58 (28) | 109 (58) | 63 (33) | 64 |
| New Territories | 22 (11) | 14 (7) | 21 (11) | 18 (10) | 11 |
| Marine and no data | 5 (2) | 6 (3) | 2 (1) | 9 (5) | 1 |

* Based on 1976 by-census.

The place of residence of both groups is given in Table II, showing that there are rather more cases from Hong Kong Island than would be expected from the population distribution of Hong Kong as a whole. This may be due to the failure to contact all the cases that occurred, and that we were more successful in Hong Kong Island than in Kowloon. This is partly due to the location of the majority of the hospitals we visited on Hong Kong Island. The controls, especially the men, are even more biased towards Hong Kong Island. In 1976, 24% of the Hong Kong population lived in Hong Kong Island, compared with 30% female cases, 52% female controls, 33% male cases and 62% male controls. This means that we must be cautious about coming to any conclusion about the distribution of cases within Hong Kong as a whole.

As expected, there was evidence of considerable mobility, 47% of the female patients and 47% of the controls having changed their place of residence in the past 10 years. For the males, 54% of the patients had moved but only 32% of the controls. This mobility makes identification of any local causal factor difficult in a chronic disease of this nature.

Cases and controls were also asked on which floor they lived now and 10 years ago. About 10% now live on higher floors than they did 10 years ago. (This is to be expected as buildings in Hong Kong are becoming taller). No other consistent difference was noted between cases and controls, either male or female.

The educational standard attained was used as a measure of socio-economic group; cases and controls were similar, and both are of a rather lower educational standard than Hong Kong residents over 45 years of age. This is to be expected, as they were all patients from general hospital wards.

Questions on cooking practice to females, both patients and controls, revealed a definite pattern. In the past wood had been the usual fuel, but in recent years a change had been made to a "modern" fuel, usually kerosene. Comparison between the female cases and the controls showed that 43 out of 189 cases never cooked and 60 cooked without kerosene; of the controls 42 never cooked but rather more (78) did not use kerosene. The average number of years of cooking with kerosene by those who used it was nearly the same, about 16 years for cases and 14·1 for controls.

Patients and controls were all asked about their occupations, and the number of years that they had been employed in each of them. They were classified according to the Hong Kong 1971 census, which is based on the

TABLE III.—*Place of origin: cases and controls*

| | Male | | | | | Female | | | | | | | | |
|---|---|---|---|---|---|---|---|---|---|---|---|---|---|---|
| | | | | | | | | | | | Non-smoking | | | |
| | Cases | | Controls | | *General population 40+ (%) | Cases | | Controls | | *General population 40+ (%) | Cases | | Controls | |
| | No. | (%) | No. | (%) | | No. | (%) | No. | (%) | | No. | (%) | No. | (%) |
| Cantonese | 174 | (83·7) | 167 | (81·9) | 83 | 166 | (87·8) | 144 | (76·2) | 84 | 74 | (88) | 104 | (75) |
| Chiu Chau | 14 | (6·7) | 20 | (9·8) | 8·4 | 10 | (5·3) | 21 | (11·1) | 7·7 | 5 | (6) | 14 | (10) |
| Elsewhere in China | 20 | (9·6) | 16 | (7·8) | 8·1 | 13 | (6·9) | 21 | (11·1) | 8·2 | 5 | (6) | 19 | (13·6) |
| Unknown, others | | | 1 | (0·5) | | | | 3 | (1·6) | | | | 2 | (1·4) |

* Based on 1971 census.

TABLE IV.—*Types of tobacco used*

| Type, total amount and brand | | Male Cases | Male Controls | Female Cases | Female Controls |
|---|---|---|---|---|---|
| *Manufactured cigarettes only* | | | | | |
| Non-users | | 4 | 44 | 101 | 151 |
| <100 kg | Western | 9 | 15 | 36 | 15 |
| | Chinese | | 1 | 2 | 1 |
| | Both | 8 | 7 | 2 | |
| | Not known | | 1 | | |
| | Total | 17 | 24 | 40 | 16 |
| 100–199 kg | Western | 45 | 40 | 26 | 9 |
| | Chinese | 2 | 1 | | |
| | Both | 4 | 11 | | |
| | Total | 51 | 52 | 26 | 9 |
| ≥200 kg | Western | 99 | 64 | 18 | 11 |
| | Chinese | 4 | | 2 | 2 |
| | Both | 33 | 20 | 1 | |
| | Not known | | | 1 | |
| | Total | 136 | 84 | 22 | 13 |
| Total users | | 204 | 160 | 88 | 38 |
| | Total | 208 | 204 | 189 | 189 |
| *Tobacco as hand-rolled* | | | | | |
| Non-users | | 169 | 181 | 126 | 151 |
| <100 kg | | 34 | 19 | 28 | 24 |
| 100–199 kg | | 2 | 3 | 12 | 5 |
| ≥200 kg | | 3 | 1 | 23 | 9 |
| Total users | | 39 | 23 | 63 | 38 |
| | Total | 208 | 204 | 189 | 189 |
| *Both manufactured and hand-rolled cigarettes* | | | | | |
| Non-users | | 2 | 43 | 84 | 139 |
| <100 kg | | 16 | 23 | 26 | 17 |
| 100–199 kg | | 46 | 51 | 29 | 10 |
| ≥200 kg | | 144 | 87 | 50 | 23 |
| Total users | | 206 | 161 | 105 | 50 |
| | Total | 208 | 204 | 189 | 189 |
| *Other forms of smoking* | | | | | |
| Non-users | | 205 | 201 | 148 | 166 |
| Users | | 3 | 3 | 41 | 23 |
| | Total | 208 | 204 | 189 | 189 |

International Standard Classification of Occupation. There was little difference between the cases and controls. The figures for the whole working population of Hong Kong according to the 1971 census are very similar to our controls.

The specific occupations recorded in the questionnaires did not reveal any particular occupation associated with an increased risk.

The places of origin of the cases and controls are shown in Table III for comparison with those of the general population of Hong Kong, based on the 1971 census of persons aged over 40 years. The controls are reasonably representative of the population of Hong Kong. There is some suggestion that the Cantonese have more bronchial cancer than the other groups. Only 4 male cases, 3 controls, 4 female cases and 5 controls had arrived in Hong Kong since 1972.

Detailed questions about smoking history aimed at finding out how much was smoked and for how long. The types of tobacco smoked were manufactured cigarettes, either of Chinese or Western origin, or "tobacco". The latter term usually refers to Chinese tobacco, which is hand wrapped in paper and smoked more or less like a cigarette. A small number smoked tobacco in other forms.

The quantity smoked in a lifetime was calculated by assuming that a cigarette contained 1 g of tobacco, the figure used in the Singapore survey (MacLennan *et al.*, 1977).

Table IV shows that the men smoked mostly Western-manufactured cigarettes. The women also smoked a few Chinese-manufactured cigarettes, but almost as many smoked hand-wrapped Chinese tobacco cigarettes as those who used manufactured cigarettes. Sixty-four women smoked tobacco in other ways, mainly the water pipe used by elderly women.

To check whether the smoking habits of the controls were typical of the whole Hong Kong population, comparison was made with the results of a survey of "Biosocial Habits" carried out in Hong Kong during 1974–75 by a team from the Australian National University (Millar, S. E., 1976, personal communication). Interviewers visited houses in Hong Kong and asked a wide variety of questions to adults in households randomly selected with the help of the Hong Kong Department of Census and Statistics. The pattern of smoking is similar between the biosocial group and our controls though the amount smoked is higher in our controls. This may be due to the younger age group surveyed in the biosocial survey, or to the lower social class of our controls.

There is certainly no evidence that we have underestimated the smoking habits of our controls; this perhaps supports the belief that there was little tendency for smokers to deny the habit. In view of the preponderance of Hong Kong residents in the controls, we checked the smoking habits of both cases and controls, male and female, in Kowloon and Hong Kong Island; no difference was apparent.

### RESULTS

The results are based on 208 male patients, 204 male controls, 189 female patients and 189 female controls.

### *Smoking*

Smoking, measured by the total quantity of cigarette or other tobacco smoked up to the time of interview, was classified as follows:

1, Non-smoker; 2, Light smoker (less than 54·7 kg, which is equivalent to 15 cigarettes a day for 10 years); 3, Moderate smoker (54·71–100 kg), 4, Heavy smoker (100·01–200 kg); 5, Very heavy smoker (>200 kg). The method of calculating the amount of tobacco smoked and the types of cigarette have been described above.

The results in Table V show a relationship between smoking and bronchial cancer, in that there were 20% less non-smokers of tobacco of any form and 24% more heavy and very heavy smokers in the cases than controls in men. In women the corresponding figures were 30% less and 24% more. However, there were among the female cases 44% non-smokers and 7% light smokers.

For the men there is very little difference between "manufactured cigarettes only" and "any forms of tobacco", the additional tobacco smoked in forms other than manufactured cigarettes increases the number of "very heavy smokers" and decreases the number of "heavy smokers" by a few percent for both the cases and the controls. For women the number of non-smokers is increased by about 10% by excluding forms of tobacco other than manufactured cigarettes, and the number of heavy smokers is decreased by about 50%. These differences apply equally to the female cases and controls.

### *Cell type*

The relation between smoking habit and

Table V.—*Smoking history: cases and controls*

| | Manufactured cigarettes only | | | | | | | | All forms of tobacco | | | | | | | |
|---|---|---|---|---|---|---|---|---|---|---|---|---|---|---|---|---|
| | Males | | | | Females | | | | Males | | | | Females | | | |
| | Cases | | Controls | | Cases | | Controls | | Cases | | Controls | | Cases | | Controls | |
| | No. | (%) | No. | (%) | No. | (%) | No. | (%) | No. | (%) | No. | (%) | No. | (%) | No. | (%) |
| Non-smoker | 4 | (1·9) | 44 | (21·6) | 101 | (53·4) | 151 | (79·9) | 2 | (1) | 43 | (21·1) | 84 | (44·4) | 139 | (73·5) |
| Light smoker | 7 | (3·4) | 10 | (4·9) | 20 | (10·6) | 4 | (2·1) | 7 | (3·4) | 10 | (4·9) | 13 | (6·9) | 7 | (3·7) |
| Moderate smoker | 10 | (4·8) | 14 | (6·9) | 20 | (10·6) | 12 | (6·3) | 9 | (4·3) | 13 | (6·4) | 13 | (6·9) | 10 | (5·3) |
| Heavy smoker | 51 | (24·5) | 52 | (25·5) | 26 | (13·8) | 9 | (4·8) | 46 | (22·1) | 51 | (25·0) | 29 | (15·3) | 10 | (5·3) |
| Very heavy smoker | 136 | (65·4) | 84 | (41·2) | 22 | (11·6) | 13 | (6·9) | 144 | (69·2) | 87 | (42·6) | 50 | (26·5) | 23 | (12·2) |
| Total | 208 | | 204 | | 189 | | 189 | | 208 | | 204 | | 189 | | 189 | |

TABLE VI.—*Cell type of carcinoma and smoking habit*

| | Male | | | | | | | | | | Female | | | | | | | |
|---|---|---|---|---|---|---|---|---|---|---|---|---|---|---|---|---|---|---|
| | Cases | | | | | | | | | | Cases | | | | | | | |
| | I+II | | III+IV | | Mixed I&III | | Unspecified | | Controls | | I+II | | III+IV | | Unspecified | | Controls | |
| Type | No. | (%) | No. | (%) | No. | (%) | No. | (%) | No. | (%) | No. | (%) | No. | (%) | No. | (%) | No. | (%) |
| All forms of tobacco | | | | | | | | | | | | | | | | | | |
| Non-smoker | 2 | (1·7) | | | | | | | 43 | (21·1) | 19 | (30·2) | 40 | (58·8) | 25 | (43·1) | 139 | (73·5) |
| Light and moderate smoker | 8 | (6·7) | 5 | (9·0) | 1 | (25) | 2 | (6·2) | 23 | (11·1) | 12 | (19·0) | 8 | (11·8) | 6 | (10·3) | 17 | (9·0) |
| Heavy smoker | 24 | (20·8) | 16 | (28·6) | 2 | (50) | 4 | (12·5) | 51 | (25·0) | 10 | (15·8) | 6 | (8·8) | 13 | (22·4) | 10 | (5·3) |
| Very heavy smoker | 82 | (70·8) | 35 | (62·5) | 1 | (25) | 26 | (81·3) | 87 | (42·6) | 22 | (35·0) | 14 | (20·6) | 14 | (24·1) | 23 | (12·2) |
| Total | 116 | | 56 | | 4 | | 32 | | 204 | | 63 | | 68 | | 58 | | 189 | |
| Manufactured cigarettes | | | | | | | | | | | | | | | | | | |
| Non-smoker | 4 | (3·4) | | | | | | | 44 | (21·6) | 26 | (41·3) | 44 | (64·7) | 31 | (53·4) | 151 | (79·9) |
| Light and moderate smoker | 8 | (6·9) | 6 | (10·7) | 1 | (25) | 2 | (6·3) | 24 | (11·8) | 18 | (28·6) | 13 | (19·1) | 9 | (15·5) | 16 | (8·4) |
| Heavy smoker | 26 | (22·4) | 17 | (30·4) | 2 | (50) | 6 | (18·7) | 52 | (25·5) | 11 | (17·4) | 4 | (5·9) | 11 | (19·0) | 9 | (4·8) |
| Very heavy smoker | 78 | (67·2) | 33 | (58·9) | 1 | (25) | 24 | (75·0) | 84 | (41·2) | 8 | (12·7) | 7 | (10·3) | 7 | (12·1) | 13 | (6·9) |
| Total | 116 | | 56 | | 4 | | 32 | | 204 | | 63 | | 68 | | 58 | | 189 | |

cell type of cancer is shown in Table VI. For the men there are about twice as many Types I+II as III+IV, but there is no indication that the type of cancer depended on the amount smoked. A high proportion of the men with cancer are either heavy or very heavy smokers, and it is clear that the patients with Types III+IV smoke more than the controls. If there was no relationship between Types III+IV and smoking, one would have expected a higher proportion of men with Types III +IV to be non-smokers, similar to that of the controls. There are no non-smokers among those with Types III+IV carcinomas and only 3 light smokers. The inclusion of other types of tobacco other than manufactured cigarettes hardly affects the results. Four men had tumours showing characteristics of both Types I and III. These different types of cells appeared in the same tumour and even in the same section. They were not 2 separate tumours occurring in the same patient.

For the women the results are rather different. There are many more non-smokers both in the cases and the controls. Forty of 68 cases of Types III+IV occurred in women who had never smoked. The smoking habits of those with Types III+IV were closer to the controls' than to those of the women with Types I+II (Table VI). It should be noted however, that 19/63 women with Types I+II never smoked tobacco in any form, and 26 smoked no manufactured cigarettes.

Table VII shows clearly an increased relative risk for Type I+II carcinomas but only a slight non-significant increase for Type III. In view of their doubtful relation to smoking the 4 patients with Type IV have been omitted: 2 were non-smokers and 2 moderate smokers. Relative risk for male patients have not been calculated, for reasons discussed below.

The smoking habits of the female cases without histological or cytological data appear to fall between Types I+II and Types III+IV (Table VI). This suggests

TABLE VII.—*Relative risk of bronchial cancer in women by cell type and smoking habit*

| | Manufactured cigarettes | | | | | | | All forms of tobacco | | | | | | |
|---|---|---|---|---|---|---|---|---|---|---|---|---|---|---|
| | Types I+II | Rel. risk | Type III | Rel. risk | All cases | Rel. risk | Control | Types I+II | Rel. risk | Type III | Rel. risk | All cases | Rel. risk | Control |
| Non-smoker | 26 | 1 | 42 | 1 | 101 | 1 | 151 | 19 | 1 | 38 | 1 | 84 | 1 | 139 |
| Light and moderate smoker | 18 | 3·6 | 11 | 1·87 | 40 | 1·78 | 16 | 12 | 3·44 | 7 | 1·36 | 26 | 1·61 | 17 |
| Heavy smoker | 11 | 3·74 | 4 | 1·41 | 26 | 1·85 | 9 | 10 | 4·16 | 6 | 1·75 | 29 | 1·97 | 10 |
| Very heavy smoker | 8 | 2·59 | 7 | 1·61 | 22 | 1·57 | 13 | 22 | 4·07 | 13 | 1·68 | 50 | 1·82 | 23 |
| All current smokers | 37 | 3·36 | 22 | 1·69 | 88 | 1·74 | 38 | 44 | 3·89 | 26 | 1·59 | 105 | 1·80 | 50 |
| Total | 63 | | 64 | | 189 | | 189 | 63 | | 64 | | 189 | | 189 |
| Trend* | $P<0·00001$ | | NS | | $P<0·00001$ | | | $P<0·00001$ | | NS | | $P<0·00001$ | | |

* Significance level 1%.

that there was no bias towards any particular histological type in this group.

Comparison made between the ratio of Types I+II to Types III+IV at different ages suggest that Types III+IV carcinomas occurs more frequently in younger men. Under 50 years of age there were 10 cases of Types I+II and 10 of Types III +IV; over 60, the figures were 61 and 19 respectively. In women the ratio was about the same at all ages.

*Bronchial cancer in women who do not smoke*

One of the main objectives of the survey was to study the occurrence of bronchial cancer in Hong Kong women who do not smoke. In this series nearly half of the women with cancer and two thirds of those with Types III+IV carcinomas were non-smokers.

Two possibilities need to be considered. Were they really non-smokers, and was the diagnosis correct?

It has been suggested that some women are ashamed to admit to smoking. It seems unreasonable to suppose that those with Types III+IV carcinomas are less willing to admit to smoking than those with Types I+II. As the proportion of those claiming to be non-smokers is higher in those suffering from Types III+IV carcinomas, either these women are less ready to admit smoking or they are in fact non-smokers. It is inconceivable that a certain proportion of our smoking patients refused to admit to smoking and that this group includes all the Types I+II patients.

As a further check, 5 female patients who had Types I+II carcinomas and said that they did not smoke were contacted on a second occasion, and their relatives were also questioned. One relative informed us that a few hand-wrapped Chinese tobacco cigarettes were smoked by a patient for a year at the age of 71. All the others continued to deny smoking, and their relatives agreed.

The other possibility is that the diagnosis of cell type is incorrect. The basis for the diagnosis of the cancers in the non-smoking women is in Table VIII, which shows that diagnosis was made largely on histological rather than cytological grounds. There is no reason to doubt either the presence of genuine cancer or that the preponderance of Types III and IV carcinomas is due to less accurate methods of diagnosis.

There is also the possibility that some of the adenocarcinomas were secondary tumours from an occult primary carcinoma of the stomach, pancreas or other site, and this could only be excluded by a full necropsy. This possibility of misdiagnosis was borne in mind when the cases were reviewed. If there was any doubt on histo-

TABLE VIII.—*Basis of diagnosis non-smoking female cases*

| | Total | Clinical | X-ray | Sputum (cytology) | Effusion (cytology) | Histological: Bronchial biopsy | Lung biopsy | Resection of lung | Lymph node | Pleural section |
|---|---|---|---|---|---|---|---|---|---|---|
| I+II | 19 | | | | 1 | 5 | 3 | 4 | 6 | |
| III+IV | 40 | | | 8 | 6 | 1 | 4 | 16 | 4 | 1 |
| Unspecified | 25 | 9 | 6 | 6 | 2 | | | 1 | 1 | |
| | 84 | | | | | | | | | |

logical or clinico-radiological grounds, the case was excluded. More important is the fact that during the period of the study there has been no reported increase in incidence of primary adenocarcinomas at other sites. Consequently, we can safely presume that the large majority, if not all, of the bronchial adenocarcinomas diagnosed in this study were primary tumours.

In order to get some indication of other possible causal factors, the following studies were made of non-smoking women with cancer: age, occupation, education, marital status, age at first marriage, number of children, place of origin, and place of residence (including floor). There was some difference in the age distribution but it was inconsistent; there appeared to be more non-smoking women over 70 years of age. The non-smoker female patients were distributed as follows: under 50—22%, 50 to 59—22%, 60 to 69—22% and over 70—34%. It is slightly different when compared with the female controls: under 50—20%, 50 to 59—30%, 60 to 69—21%, and over 70—28%.

For the other factors no difference could be detected. If anything, the non-smoking females, both cases and controls, lived on slightly lower floors than the smokers.

Table IX shows that more of the female cases, including non-smokers, used kerosene or gas for cooking than the relevant controls. For all female cases the average number of years of using kerosene was 16 years and for controls 14·1 years, for non-smoking women the figures were the same. On the other hand, 15 women with cancer who did not smoke stated that they did not cook at all.

The answer to the question "Are you exposed to the tobacco smoke of others at home or at work?", gave no indication that other people smoking was a contributory factor in the non-smokers. Forty per cent of non-smoking women with bronchial cancer complained of such exposure as compared with 47% of non-smoking controls. It must be admitted that this is a rather subjective approach to the problem.

DISCUSSION

*Association between smoking and bronchial carcinoma*

There have been many case-control studies in many parts of the world which showed a relationship between smoking and bronchial cancer, including one in Hong Kong by Leung (1977). As our study reveals nothing new in this respect, little space will be devoted to this part of the discussion. We confirmed a relationship between smoking and bronchial cancer in both sexes. Comparing smokers with non-smokers in cases and controls shows a high

TABLE IX.—*Female cooking habits*

| | All women: Total | Never cook No. (%) | Never cook with kerosene No. (%) | Never cook with kerosene or gas No. (%) | Non-smokers: Total | Never cook No. (%) | Never cook with kerosene No. (%) | Never cook with kerosene or gas No. (%) |
|---|---|---|---|---|---|---|---|---|
| Cases | 189 | 43 (22·8) | 60 (31·7) | 54 (28·6) | 84 | 15 (22·4) | 23 (27·4) | 18 (21·4) |
| Controls | 189 | 42 (22·2) | 78 (41·3) | 62 (32·8) | 139 | 29 (20·0) | 56 (40·3) | 43 (30·9) |

level of significance for men ($\chi^2=45$ $P<0{\cdot}01$) and some significance for women ($\chi^2=4{\cdot}3$, $0{\cdot}05>P>0{\cdot}02$). An increased relative risk has been shown for women (Table VII). With so few male non-smokers we did not think it justifiable to present the relative risks for men.

We have shown that in age and educational level the controls are similar to the cases. In one respect there is a dissimilarity: a higher proportion of the controls live in Hong Kong Island. But we have confirmed that there is no difference in smoking habits between those living in different parts of Hong Kong.

A criticism has been made of the use of orthopaedic patients as controls. It is suggested that smoking results in osteoporosis and that therefore patients in orthopaedic wards may be suffering from smoking-related diseases (Noel Weiss, 1978, personal communication). The comparison of our results with that of the bio-social survey noted above, and our use of orthopaedic patients, both suggest that, if anything, our controls smoke rather more than a group really representative of the population of Hong Kong. This would tend to minimize the difference in smoking habit between the cases and the controls and tend to obscure a real relationship.

The conclusion seems clear that in Hong Kong as elsewhere there is a relationship between smoking and bronchial cancer, certainly in the particular social group that we have been studying, and that in Hong Kong, as elsewhere, reduction of smoking will reduce the incidence of this disease. However, this will clearly not apply to the women who do not smoke. Study of this group will help to reveal other causal factors.

*Histological type*

An important objective of this study was to try to relate the cell type of the tumours to smoking habit.

In comparison to European series the proportion of adenocarcinoma has been high in Hong Kong (Chan & MacLennan, 1977) and also in Singapore (Law *et al.*, 1976). In our series it amounted to 26% in men and 49% in women. Chan & MacLennan (1977) have shown that there has been no tendency for the proportion of adenocarcinoma to decline during the period of rapid increase in incidence of bronchial cancer as a whole in Hong Kong. It is therefore reasonable to assume that exposure to factors causing adenocarcinoma has also been increasing.

Previous reports of the relationship of Type III carcinoma to smoking have been conflicting. Wynder & Hechts (1976) found a strong association between smoking and tumours of Kreyburg Group I in American women, but Kennedy (1973) could detect no such association in Britain.

Before discussing the results of the histological findings in this series it is necessary to examine the methods and material on which they were based. The sources of information have been given above in Table I. Specimens from 93 women, including 38 Type III carcinoma, were diagnosed histologically, and from 60 women, including 26 Type III carcinoma, by cytology. Twenty-two of the histologically diagnosed Type III carcinomas were based on resection specimens.

If we accept that reliance can be placed on the diagnosis, there is a clear relationship between smoking and cell type in women (Table VII). A definite increased relative risk has been shown for women with Types I+II (3·89), but only a small increase (of doubtful significance) for Type III (1·59).

In the men it is not possible to detect any suggestion that one particular type of tumour is associated with smoking.

*Bronchial cancer in women*

Bronchial cancer now causes about a quarter of the deaths of females from cancer in Hong Kong, and the incidence is rapidly increasing. There were 50 female deaths from bronchial cancer registered in 1956, 248 in 1966 and 532 in 1976. Although part of this increase may be due to improved diagnosis, and to a larger number of women in the older age groups, the disease is now a formidable problem, and not merely of the very old. Of 189 women in our series, 24 were below 50 years of age and 47 between 50 and 60. Reduction of smoking will not prevent this disease completely. Half of the women in our series did not smoke, and some of the tumours in smokers are likely to be due to causes other than smoking.

It is necessary to search for a cause of this cancer that has been increasing over the past 20 years and that tends to produce adenocarcinoma. The factors considered in this study were occupation, air pollution and fuel used for cooking.

The study of occupation in Hong Kong is not easy, as there has been great mobility over the past 20 years. Certainly our questions did not suggest any particular occupation as being dangerous. The majority of the women, both cases (63%) and controls (68%) were involved in housework, either in their own homes or in other people's houses as amahs.

The possibility of air pollution as a cause of bronchial cancer has recently been reviewed by Higgins (1976). He considered benzpyrene and sulphur compounds. There are no figures for air pollution by benzpyrene in Hong Kong, although it is probably increasing, but it is unreasonable to think that it is higher than in other industrial cities which do not have this high incidence of bronchial cancer in non-smoking women.

Information is available about sulphur dioxide pollution in Hong Kong which shows that there have been high levels of sulphur dioxide in the past in one particular area of Hong Kong (Hung Hom) from an electricity generating station with low chimneys because of its proximity to the airport runway. Records kept by the Air Pollution Control Unit of the Hong Kong Labour Department showed that in 1967 there were high levels of sulphur dioxide, varying from a monthly average of 51·4 to 116·8 parts/$10^8$ during the year at Hung Hom. In other parts of Hong Kong the level was practically always below the recommended upper level of 8, and usually below one. There has been a gradual improvement in Hung Hom, and by 1976 the monthly average never exceeded 3, and the figures for other parts of Hong Kong remained low (Hong Kong, 1967–76). Correlation of the incidence of cancer with this source of pollution is not easy, because of the mobility of the population in the years before diagnosis, and also because of the biased selection of our cases with regards to place of residence. However, the observed distribution of patients in Kowloon, when compared with that expected if they had been distributed merely according to population density, showed no clustering of cases, and there was no excess in and around the Hung Hom power station.

Correlation with level of residence revealed no pattern, and it was suggested (K. W. Fung, 1978, personal communication) that there is no consistency about sulphur dioxide concentration on different floors in blocks of flats. In some parts of Hong Kong it is higher on the upper floors and in others on the lower. Our findings do not support the theory that air pollution could be the cause of bronchial cancer in women who do not smoke. Again, we should need to explain why such pollution has such a marked effect in Hong Kong, compared with other parts of the world.

It has been suggested that the Southern Chinese system of cooking may have some relation to the development of lung cancer. Cantonese cooking requires a higher temperature in the pan than the methods used in other provinces of China. It is suggested that products of the heated cooking oil contain carcinogens such as nitrosamines and that these have increased over recent years owing to the change from firewood to kerosene, which has a hotter flame, or to the use of less pure oils which produce more nitrosamines. In Cantonese cooking the pan does become hotter before the oil or the food is introduced. This may lead to greater decomposition of oil and foodstuffs.

Leung (1977) has reported that women in Hong Kong with bronchial cancer cook more frequently with kerosene, though it seems that the controls he used were not strictly comparable to his cases.

There is no strong evidence to suggest that lung cancer is related to cooking with various kinds of fuel in either smoking or non-smoking women. In our series fewer women with cancer "never cook with kerosene or gas", but the difference was small. When non-smoking women patients were compared with non-smoking controls there was no significant difference ($\chi^2 = 1{\cdot}92$; $0{\cdot}1 < P < 0{\cdot}2$).

Probably more noteworthy is that, of the 84 female non-smokers with bronchial cancer, 23 claimed they had never cooked with kerosene, 18 never cooked with kerosene or gas and 15 never cooked at all.

As in the argument that cancer in non-smokers cannot be due to smoking, it is difficult to attribute cancer in those who do not cook to the fumes in the kitchen. These arguments are not conclusive, and perhaps the whole question of carcinogens from cooking should be further investigated, including a more detailed investigation into cooking habits. One may doubt whether so many Hong Kong women really do not cook. It is worth noting that equal numbers of "professional cooks" were found in cases and controls in both males and females.

Two other possible causes have been suggested but were not considered in this study. The relationship of bronchial cancer with previous tuberculosis has been discussed by Steinitz (1965) and is being investigated in Hong Kong by Mok (1977, personal communication). In Japan there is evidence that bronchial cancer is commoner in those with a diet deficient in yellow-green vegetables (Hirayama, unpublished). It is not easy to see why either of these factors should apply particularly to Cantonese women, whose cooking methods preserve vitamins in vegetables.

The search for a cause of bronchial cancer in women who do not smoke certainly demands further study.

We wish to express our gratitude to three groups of people whose help and cooperation made this study possible.

The International Agency for Research on Cancer for partial financial support, and especially to Dr R. MacLennan who visited Hong Kong twice and gave us very great help both in the planning and data processing.

The consultants at the hospitals who allowed us to interview their patients: Dr W. S. Low at Kowloon Hospital, Sister Mary Aquinas at Ruttonjee; Dr Rudy K. K. Khoo at Queen Mary Hospital, Drs S. M. Teh and Y. F. Poon at Queen Elizabeth; at Grantham Hospital, first Dr P. A. L. Horsfall and later Dr C. K. Mok; and Professor A. M. C. Yau and Dr P. C. Leung who allowed us to interview the orthopaedic patients who formed the controls.

Special thanks are due to Mrs J. Cheang, Field Nurse in the Department of Community Medicine, who interviewed all the female patients and controls, to Mrs T. Lam and Mrs P. Liu for secretarial help and Mr C. L. Chan for statistical assistance.

### REFERENCES

CHAN, W. C. & MACLENNAN, R. (1977) Lung cancer in Hong Kong Chinese: mortality and histological types, 1960–1972. *Br. J. Cancer*, **35**, 226.

COLBOURNE, M. J. (1976) Mortality trends in Hong Kong. *Community Medicine, Hong Kong*. **8**, 18.

GILBY, E. D. (1978) Diseases of respiratory system: Neoplasms of lung. *Br. Med. J.*, i, 1331.

HIGGINS, I. T. T. (1976) Epidemiological evidence on the carcinogenic risk of air pollution. *INSERM Symposia Series, 52, IARC Scientific Publication No. 13.*

HONG KONG (1967–76) *Annual Reports of the Labour Department*. Hong Kong Government Printer.

HONG KONG (1972) *Hong Kong population and housing census 1971 main report*. Census and Statistics Department, Hong Kong Government Printer.

HONG KONG (1977) *Ann. Rep. Medical & Health Department*. Hong Kong Government Printer.

KENNEDY, A. (1973) Relationship between cigarette smoking and histological type of lung cancer in women. *Thorax*, **28**, 204.

LAW, C. H., DAY, N. E. & SHANMUGARATNAM, K. (1976) Incidence rates of specific histological types of lung cancer in Singapore Chinese dialect groups and their aetiological significance. *Int. J. Cancer*, **17**, 304.

LEUNG, J. S. M. (1977) Cigarette smoking, the kerosene stove and lung cancer in Hong Kong. *Br. J. Dis. Chest*, **71**, 273.

MACLENNAN, R., DA COSTA, J., DAY, N. E., LAW, C. H., NG, Y. K. & SHANMUGARATNAM, K. (1977) Risk factors for lung cancer in Singapore Chinese, a population with high female incidence rates. *Int. J. Cancer*, **20**, 854.

ROSE, R. J. (1960) *Maori–European standards of health*. New Zealand Department of Health special report, series 1.

STEINITZ, R. (1965) Pulmonary tuberculosis and carcinoma of the lung. *Ann. Rev. Resp. Dis.*, **87**, 758.

WATERHOUSE, J., MUIR, C., CORREA, P. & POWELL, J. (Eds). (1976) *Cancer incidence in five continents*, V. III. Lyon: IARC Scientific Publication No. 15.

WYNDER, E. L. & HECHTS, S. (Eds). (1976) *Lung Cancer*. Geneva: UICC.